# Dr. Merriman’s Address

**Published:** 1869-05

**Authors:** 


### CHICAGO MEDICAL EXAMINER.

N. S. DAVIS, M.D., EDITOR.

VOL. X. MAY, 1869. NO. 5.

### Original Contributions.

ARTICLE XVII.

### DR. MERRIMAN'S ADDRESS.

DELIVERED TO THE ALUMNI OF THE CHICAGO MED. COLLEGE.

BROTHER ALUMNI:

The constitution of our Society says, its first object shall be "to keep alive and perpetuate that kind and cordial feeling which binds us together by reason of our common *Alma Mater*"—which I understand to mean that we shall assemble here to have a good time; that we shall lay aside our pills, powders, and prescriptions, our saws, splints, and scalpels, our grave faces, and wise looks, and feel that we have so far conquered success, that we can afford for to-day to look around us and see what work we have done, not only individually, but collectively; that we can grasp each other's hands with hearty friendliness and congratulations upon the many successes the Alumni have had.

It is very pleasant to meet together as we are doing to-day—to see that *Alma Mater* is growing, as we perceive from the accession to our ranks of so fine a class as will to-morrow receive their diplomas—to meet many of our old associates, and to hear from others—how some of them are across the great waters, and some about to go, to pursue the same studies they commenced here so thoroughly—how others are winning forthemselves greenbacks, or laurels, or, better still, the love and gratitude of whole communities; so that to lose their doctor, or have any evil befall him, would be looked upon not only as a public, but, to each individual, a private loss.

Thus, the sons of our *Alma Mater* are growing in numbers, wisdom, and power; and the dear old Mother herself is not standing still. Within the last two years, four of her original faculty have added the value of an European experience to their previous thorough acquisitions, while three more have come to us fresh from those time-honored schools: and, not content with her previous requirements, *Alma Mater* is the first of American colleges to demand of her students three courses of lectures, and six months in each course, instead of the customary two courses, four months each, of sister institutions. With her progressive system, giving an entirely new course each year of the three, *our* college goes over nearly twice the ground of any similar institution in the country. The system adopted here is attracting great attention in Eastern cities. Professor Bumstead, in the "Introductory Lecture," at the College of Physicians and Surgeons, last fall, advised the students to choose from the course there given anatomy, physiology, chemistry, and materia medica, and to pay their attention chiefly to those studies the first year, and to the others the second year. Seeing, then, what she has done, and is doing, how proud we are of *Alma Mater* to-day; and I feel that each year as we assemble here we shall have new and greater cause for so pardonable a pride.

While we thus meet to revive old friendships and recollections, and to enjoy the social hour together, we ought not to forget to take home with us new determinations to work for the exhaltation of our noble profession, and to increase its usefulness. The success of one is an honor to all of us. Each new truth developed or old one made clear adds to the power of every physician, and gives the profession a stronger claim to the love and respect of the human race. Science has a claim upon each one of her votaries; and, as Sparta demanded that each subject should be a brick in her strong wall of defence, so the great Genius of Medicine may say to each one of us,—"You must give me strength, or I shall become feeble."

Though some men have gone forth from our *Alma Mater* who are already wielding an influence, not only in their own locality, but upon science everywhere—still, our college is not old—few can *look back* to famous deeds; but the future opens to us, and—

> "As drops of rain fall into some dark well,
> And from below comes the scarce audible sound,
> So fall our thoughts into the dark hereafter;
> And the mysterious echo reaches us."

But those delightful echos from the unseen future, those dreams of high achievements, fame, and happiness, are not separated from us merely by a space of time; we cannot sit down at ease with any prospect of their being realized, nor will accident give them to any of us. Accident *sometimes* gives men a discovery which benefits the world; but, as a rule, the accident is merely the suggestion of a most thoughtful mind. The discovery of the pendulum and of the steam-engine required minds that could work out a great principle from events so common as to be scarcely noticed by ordinary men. Apples had fallen before human eyes for thousands of years before a mind like Newton's seized the suggestion, and deduced from it that wonderful law which binds the planets to the sun, and the great systems all in one.

We may safely lay it down, as a rule, that no man will accomplish anything to-morrow who does not accomplish something to-day. The surgeon who, in the future, will most creditably perform the great capital operations is he who, in the present, is most diligently preparing himself. In such an operation, the important moment to the surgeon is not when he takes his knife in hand, with the anæsthetized patient before him, but, rather, when in previous study he has gone over, *again and again*, every step of the operation, till he is as sure of it as the penman of his power to form a letter. If, then, we look for the causes of success, we shall find them in the early lives of successful men. Let us take a few examples of such men, and see if we cannot draw from their early lives a lesson which, if applied to our own, will help us on toward the career we all desire. Demosthenes, the orator, inherited a most delicate constitution; but he overcame the defect by the most rigid temperance in food and drink, conjoined with a thorough system of exercise. He was troubled with a stammering speech and shortness of breath, which he overcame by the most incredible labor and perseverance. He shut himself up, for months at a time, to practice his beloved art, and copied the lengthy works of a noted author, whose style he admired, no fewer than eight times.

What American is not proud of the career of Benjamin Franklin, the printer boy, who became the eminent statesman? His father frequently repeated to him in boyhood:—"Seest thou a man diligent in his business, he shall stand before kings." He said he was reminded of this when at the court of France. His diligence in making opportunities, and in improving them when made, are too well known to you to need repetition. The man who could live upon saw-dust pudding and water *did not* need any man's patronage.

Sir Astley Cooper, though a wild and ungovernable boy, became, at the age of sixteen, a most indefatigable student of medicine. His diligence in anatomy was so great that he stood at the head of the class in the beginning of his second year; and, before he was eighteen, he was admitted to membership in the Physical Society—the oldest medical society in London. Entirely reversing his former habits, he spent his vacations in the closest study. He early became a teacher, and such was his professional zeal that, even on the day of his marriage, he gave his lecture in the evening, as usual. What result but success could follow such devoted labors?

Space permits me to cull merely a suggestion from these noted examples; but, through all the thousands that history records, we find this same precept enforced—"The hand of the diligent maketh rich."

I cannot resist the desire to mention one more example—that of one whom we have all learned to love for his largeheartedness, and revere for his intellectual power and great accumulations of medical knowledge. It is unnecessary for me to mention a name; you will all recognize the man.

In early life poor, having hardly what is now considered a common school education, without influential friends, without opportunities, except such as he made himself, with, absolutely, no advantages, save a soul endowed with a determination *to do*, he began his career. Soon after, he graduated, the Medical Society, of New York, offered a prize for the best essay on "Diseases of the Spinal Column." He knew no more of the subject than the most recent graduate, but at once began to study it up; wrote the essay, and gained the prize. The next year, he gained another, upon "Discoveries in the Physiology of the Nervous System, since the time of Sir Charles Bell," in which were first mentioned many of the points that have since become generally accepted. Thus, he went on investigating new subjects, and writing new articles every year, always keeping on hand a list of obscure points for investigation. On removing to New York, he at once became Assistant to the Demonstrator of Anatomy, in the College of Physicians and Surgeons; and, while there, passed through the cholera epidemic of 1849. He remained but a short time in that city, and then came here. His history since, you all know—how from early morn till late at night, in storm and sunshine, in season, and out of season, he toiled and *succeeded*.

Although my description has been of only one man, he is but a type of our faculty. To their assiduous labors and enterprise, no less than to their intellect, do they owe their high standing in the profession.

Just here, the thought comes to me, "Who are are to take the places of the illustrious men of the present day, when they pass from the stage of active life?" Let each one of us ask himself—"May I not be among the number?" for each generation are offered the same prizes for the same endeavor.

The reason of most failures is, that men want a practice without earning it, and success without deserving it. He who is willing to pay the price of these things in labor and self-denial will surely gain them.

*Work*, then, first, to make yourself all you can be. Whatever else you do, don't neglect to grow. Remember that when you graduated, the close of the course was called "commencement," and not "completion." You were turned loose upon humanity as doctors; but with some little doubt as to whether you would prove destructive or not. "A little knowledge is a dangerous thing," you know; and those who rely upon what they gained at lectures only as sufficient will really prove dangerous. Our weapons are powerful for good or ill, according to the way they are used; and in an unskilful hand are always to be dreaded. I had rather trust a friend to the infinitessimals of true homœopathy than to the half-informed practitioner of the regular school; especially if he be heroic. Continue your medical studies; keep always on hand subjects for investigation, and study them; be an active member of medical societies; write for medical journals; always have a medical student, and be sure you instruct him,—for the surest way to learn a thing is to teach it.

*Work*, secondly, for influence, and then to use it aright. Physicians are becoming every year more and more men of weight in the community; and this will continue to be so as the profession becomes more advanced, and culture has more sway. Bring your influence to bear upon the prominent men, and the Legislators, that they may give correct votes on bills for legalizing dissections, and providing *materiel* therefor, also to pass laws restraining quackery, regulating practice and druggist's duties, and the sale of poisons; and urge your Medical Society to use its influence in the same direction.

Don't be discouraged at failure. The man who never failed is a myth; and, frequently, the men who have failed the most have become the most successful in the end.

It was a beautiful idea of the ancient Greeks—that classic people—to make Apollo God of medicine. Apollo, you remember, was God of the Sun—that symbol of life and peace and happiness. He was also God of music, which, among them, included all kinds of culture. Æsculapius was a kind of patron saint of physicians; but the real God of medicine was he who united in himself manly grace and beauty with genial ways and the highest culture, of all the heathen divinities, the one who would to-day be called the truest gentleman.

Let us from him, too, draw our lesson; and, with all medical knowledge, mingle the kind disposition, the agreeable manners, and the general culture of the gentleman: but let us lift ourselves higher than to be merely disciples of Æsculapius or Apolo; and, as Christianity is higher than heathenism, let us rather be followers of Him who is the grandest model of all virtues, and who alone is called the "Great Physician."

